# The key design features and effectiveness of social network interventions for HIV testing and linkage services in low‐ and middle‐income countries: a systematic review and meta‐analysis

**DOI:** 10.1002/jia2.26458

**Published:** 2025-04-25

**Authors:** Madalo Mukoka, Takondwa C. Msosa, Hussein H. Twabi, Robina Semphere, Marriott Nliwasa, Guy Harling, Alison Price, Katherine Fielding, Augustine T. Choko

**Affiliations:** ^1^ Helse Nord Tuberculosis Initiative Department of Pathology Kamuzu University of Health Sciences Blantyre Malawi; ^2^ Department of Infectious Disease Epidemiology and International Health London School of Hygiene and Tropical Medicine London UK; ^3^ Department of Global Health Amsterdam University Medical Centres Amsterdam the Netherlands; ^4^ Institute of Life Course and Medical Sciences University of Liverpool Liverpool UK; ^5^ School of Health and Wellbeing University of Glasgow Scotland UK; ^6^ Institute for Global Health University College London London UK; ^7^ Malawi Epidemiology and Intervention Research Unit Lilongwe Malawi; ^8^ Department of Population Health London School of Hygiene and Tropical Medicine London UK; ^9^ Malawi‐Liverpool‐Wellcome Clinical Research Programme Blantyre Malawi; ^10^ Department of International Public Health Liverpool School of Tropical Medicine Liverpool UK

**Keywords:** HIV care continuum, intervention, key and vulnerable populations, linkage to care, LMIC, social networks

## Abstract

**Introduction:**

HIV remains a global health challenge with a reported 39 million people living with HIV (PLHIV) in 2022. Sub‐Saharan Africa, Asia and the Pacific are home to 82% of PLHIV, where limited access to healthcare resources underscores the urgency of innovative strategies to combat the epidemic effectively. Social network interventions (SNIs) hold promise for improving HIV testing and linkage services by engaging populations at greatest risk. This review evaluates the key design features and effectiveness of SNIs for HIV testing and linkage in low‐ and middle‐income countries (LMICs).

**Methods:**

We searched four databases (Medline, Embase, Global Health, Web of Science) for the period from 1st January 2003 until 16th June 2023. A combination of the terms “Social Network,” “HIV,” “testing” and “linkage” with an LMIC filter was used. We included interventional study designs that compared an SNI for HIV testing and/or linkage to care against non‐network comparator approaches. Narrative synthesis and random effects meta‐analyses were conducted to synthesize the results.

**Results:**

Of the 6763 records, 13 studies met the inclusion criteria; eight were randomized controlled trials, and five were non‐randomized designs. Nine studies engaged key populations. The most common strategy involved recruiting and training seeds, who then delivered HIV services to network members. The use of networks varied significantly across the papers. The network approaches used were induction (*n* = 11), alteration (*n* = 1) and a combination of individual and segmentation approaches (*n* = 1). The pooled estimates showed that SNIs had a modest effect on the uptake of HIV testing RR 1.12 [95% CI 1.08−1.17) but the directionality of effect for the proportion newly diagnosed positive (RR 0.88 [95% CI 0.74−1.04]) and linkage to care (RR 0.98 [95% CI 0.86−1.08]) was towards the null.

**Discussion:**

SNIs improved the uptake of HIV testing and exhibit important variability in their design.

**Conclusions:**

There is a need for more studies designed to capture the complex relational dynamics of network interventions and to provide strong evidence on their isolated effects. Additionally, it is necessary to expand the use of network approaches to other priority populations.

**PROSPERO Number:**

CRD42023434770

## 1 INTRODUCTION

HIV remains a global health challenge with an estimated 39.9 million people living with HIV (PLHIV) in 2023 [1]. Progress towards achieving the UNAIDS 95‐95‐95 targets has been remarkable with 86% of PLHIV knowing their HIV status, 89% of PLHIV being on treatment and 93% of PLHIV on treatment being virally suppressed as of 2023 [1]. However, low‐ and middle‐income countries (LMICs) still shoulder a disproportionate burden of HIV—despite decades of investments towards eliminating the epidemic [1]. Sub‐Saharan Africa (SSA), Asia and the Pacific are home to 82% of the PLHIV globally [1]. Nevertheless, despite considerable efforts to scale up HIV testing and treatment services, gaps persist in the identification of individuals living with HIV and their subsequent linkage to care. Individual (such as costs, stigma, fear, normative gender roles), environmental (such as cultural beliefs, lack of support) and systemic factors (such as inflexible facility hours, limited access) create barriers for certain groups of people such as men, adolescents and key populations from accessing conventional facility‐based HIV testing and treatment [2, 3, 4, 5, 6]. Consequently, untreated HIV is becoming concentrated in such groups [1, 7]. Therefore, using social networks to enhance HIV testing and linkage services is a strategy with tremendous potential to improve the utilization of HIV services as well as targeting populations at greatest risk [8].

Social networks are a set of individuals or social actors and the ties among them, and they play a critical role in shaping health‐related behaviours and outcomes [9, 10]. Social ties tend to amplify information or behaviour spread such that the behaviour of one person can lead to behaviour change in the population at large [10, 11, 12]. The influence of social networks is particularly relevant in the context of HIV prevention and care. Networks can either facilitate or hinder both risky behaviours that facilitate transmission and access to HIV testing and linkage to care, through network functional aspects such as stigma, social support, information dissemination, and shared norms and attitudes [13, 14, 15].

Social network interventions (SNIs) involve using or manipulating network ties to change other outcomes [16]. Valente describes a taxonomy of SNI approaches, specifically: (1) Individual—selecting individuals to champion an intervention on the basis of some network property; (2) Segmentation—directing an intervention towards selected groups rather than individuals to maximize local uptake; (3) Induction—creating novel interactions between people already connected to one another; and (4) Alteration—interventions that change the network structure by creating or deleting people or ties [16]. These approaches underlie the varied use of networks in accelerating HIV testing, counselling and linkage to care among individuals at risk or already living with HIV [17, 18, 19].

Many variants of SNIs for HIV services exist in literature and are sometimes referred to as peer‐driven interventions, social network strategy, respondent‐driven recruitment, chain referral or secondary distribution [19, 20, 21, 22]. The use of networks equally varies; some studies use new or existing ties to enhance recruitment, disseminate HIV‐related information or to distribute HIV self‐testing (HIVST) kits in‐person or via mail and in some instances social media (virtual ties) is used as an outreach platform to reach many at once [23, 24, 25, 26]. Studies aiming to improve adherence to treatment or retention in care frequently use peer support, where a peer (known or unknown) is linked to the participant as a buddy to provide the support necessary to encourage the participant to engage in care [27].

In addition to the lack of uniform terminology to describe SNI, there is a lack of standard assessments required in network analysis and disparate descriptions of SNI in the published literature. Despite the existence of Valente's taxonomy, much of the published SNI literature for HIV services does not use the taxonomy or provide comparable information. The heterogeneity in reporting SNI studies, including the methodology used, provides a challenge to identifying, designing and implementing context‐appropriate social network approaches for the LMIC setting.

This systematic review and meta‐analysis involved identification, review and analysis of existing literature to understand the key design features, and the effectiveness of SNI for HIV testing and linkage services in LMICs. Including LMICs, despite their diversity, is crucial as they often face unique health system constraints and social dynamics that can influence the feasibility and impact of SNI. By synthesizing the findings from various studies conducted in these diverse settings, this review seeks to highlight the potential benefits, challenges and best practices associated with this approach thereby providing valuable insights into their applicability in resource‐limited settings.

## 2 METHODS

### 2.1 Search strategy and selection criteria

This systematic review was conducted and reported according to the Preferred Reporting Items for Systematic Reviews and Meta‐Analyses (PRISMA) guidelines and was registered with PROSPERO (International prospective register of systematic reviews) CRD42023434770 [28]. We searched four databases; Medline, Embase, Global Health and Web of Science for the period ranging from 1st January 2003 through 16th June 2023 to focus on the era of universal access to antiretroviral therapy [29]. A detailed search strategy can be found in Table S1. A combination of the following search terms was used: “Social Network,” “HIV,” “testing” and “linkage.” A geographical limitation was added to the search using the Cochrane's LMIC filter [30]. The search strategy was informed by previously published reviews, the authorship team and a librarian.

We included interventional study designs such as cluster randomized controlled trials (CRTs), individually randomized controlled trials (RCTs), non‐randomized trials (non‐random assignment of intervention) and quasi‐experimental studies (pre‐post or interrupted time series design) that compared an SNI for HIV testing and/or linkage to care against comparators that did not use social networks in the recruitment or delivery of HIV testing and/or linkage. Post hoc, a decision was made at the time of data extraction to include studies that had social network‐based comparator groups in addition to the comparator group without social networks. This was done to capture the broader spectrum of evidence on how SNIs perform relative to other network approaches. Participants were individuals belonging to any social network such as friends, family members, work friends, sexual partners or risk‐sharing groups (female sex workers [FSWs], men who have sex with men [MSM] or people who inject drugs [PWID]) with no age restriction.

In this review, SNIs were defined as “purposeful efforts to use social networks or social network data to generate social influence, accelerate behaviour change, improve performance, and/or achieve desirable outcomes among individuals, communities, organizations, or populations” [16]. The ties could have existed before the commencement of the intervention or could have been formed for the purpose of the intervention. We did not limit our inclusion to studies that reported ties that exist in a physical space but also included ties that exist through virtual means such as social media. Thus, we included studies that provided HIV testing and/or linkage services and used social ties to improve or accelerate the engagement of these services. This could have been achieved either through direct distribution of the service through social networks or the recruitment of participants using network techniques.

As the use of social networks varies in studies, we excluded interventions that used social media as an outreach platform only without any effort to create new ties or use existing ties to accelerate HIV testing or linkage. We also excluded interventions that used social networks descriptively, that is network characteristics were described but not used to accelerate recruitment of participants or delivery of the intervention.

### 2.2 Outcomes

We included studies that had at least one of the following four outcomes: proportion of individuals who were approached for HIV testing and subsequently underwent testing (uptake of HIV testing); proportion of individuals who underwent HIV testing and tested positive for HIV (yield); proportion of newly identified PLHIV linked to HIV care and treatment services (defined as either proportion initiating antiretroviral therapy (ART) or proportion registered entry at HIV clinic or proportion with initial viral load test or proportion with initial CD4^+^ count or proportion retained on ART) [31]; and proportion linked to preventive services (proportion initiating pre‐exposure prophylaxis [PrEP] or proportion uptake of voluntary medical male circumcision).

### 2.3 Data extraction and quality assessment

Articles identified through the search were imported into Rayyan [32] where deduplication and title and abstract screening were done. Authors (MM, TCM, HHT and RS) independently reviewed the titles and abstracts and prepared a list of all articles eligible for inclusion. Full‐text review was then performed against the eligibility criteria to remain with only those that met the criteria. Discrepancies at both stages were resolved by KF, AP, GH and ATC. A standardized form was used to extract data including authorship and date of publication, intervention description, study design, study setting, participants (inclusion and exclusion criteria), social network details (approach used, promoter identification and use of networks or network data), incentives used, outcome measures and main study findings. Authors were contacted for full‐text articles missing critical information. For trials with multiple arms, data were extracted from eligible arms only. The revised Cochrane Risk of Bias Tool (RoB 2) was used to assess the validity of RCTs (individual or cluster) and the Risk of Bias in Non‐Randomized Studies of Interventions (ROBINS‐I) was used to assess non‐randomized and quasi‐experimental studies [33, 34].

### 2.4 Statistical analysis

Included studies had outcomes relating to behaviour change towards HIV testing or linkage to care. Outcomes could have been based on objective measures or self‐report. Outcomes were compared between the SNI group and the non‐network‐based comparator (as originally planned). Studies with an SNI comparator were reported separately and not included in the meta‐analysis.

A narrative synthesis was done summarizing the study population, study design, key features of the intervention and outcomes. A meta‐analysis using the random effects model was used to pool the outcome measures and the corresponding 95% confidence interval (CI) for randomized trials (RCT or CRT). The differences between intervention and comparator arms were expressed as risk ratios (RR) and corresponding 95% CI. Statistical heterogeneity was evaluated using the *I* ^2^ statistic. A subgroup analysis using the *Q*‐statistic to investigate the effects of network intervention approaches and country income classification was conducted. Analysis was done using the meta and metafor packages in R (version 4.4.1) [35].

## 3 RESULTS

### 3.1 Study selection

**Figure 1:**
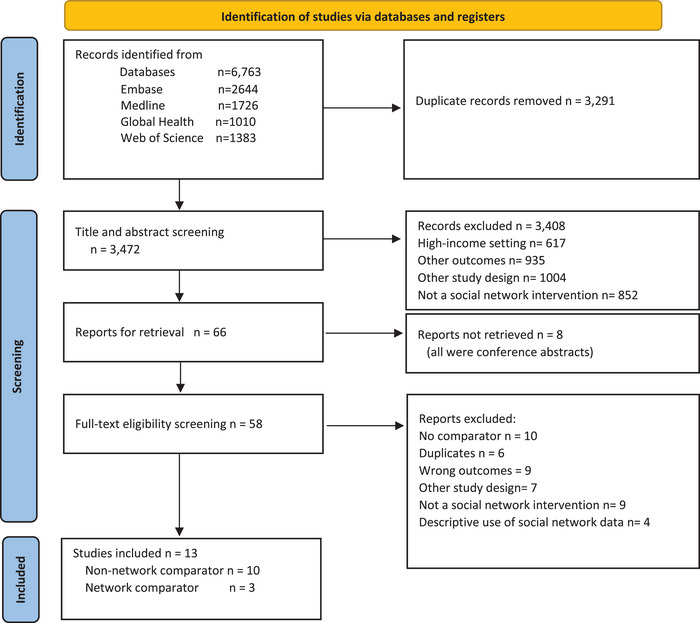
PRISMA flow diagram.

We identified 6763 records from database searching and 3472 records underwent title and abstract screening following the removal of duplicates (Figure 1). At title and abstract screening, we excluded papers that did not describe an SNI, did not involve HIV testing or linkage, employed ineligible study designs or were conducted in high‐income countries. Of the remaining 66 potentially eligible records, 8 records could not be retrieved (conference abstracts), 45 were excluded for the following reasons: had no comparator (*n =* 10), duplicates (*n =* 6), had wrong outcomes (*n =* 9), other study design (*n =* 7), did not investigate an SNI (*n =* 9) and used social network data descriptively (*n =* 4)—Figure 1. This left 13 records for inclusion in the review.

### 3.2 Study characteristics

Data were extracted from the remaining 13 records: 10 with a non‐network comparator and three with a network comparator (Table 1). Of the 13 records, five were cluster randomized studies [36, 37, 38, 39, 40], three were individually randomized studies [41, 42, 43], two were non‐randomized trials [44, 45] and three were quasi‐experimental studies [46, 47, 48]. These studies were conducted in 10 different LMICs (two low income and eight middle income) between 2015 and 2022. Most of the studies engaged key populations; MSM (*n =* 5), FSW (*n =* 2) and PWID (*n =* 2). The remaining studies were done among the general population (*n =* 4).

**Table 1:** Characteristics of studies meeting the inclusion criteria

First author, year	Country	Study design	Sample size	Population	Intervention	Comparator	Network approach	Promoter selection	Main finding^a^
**Non‐network‐based comparator**									
Pines HA, 2021	Mexico	Quasi‐experimental	2072	≥18 years old MSM or transgender female	Recruitment coupons to social networks of MSM and TW	Targeted recruitment and testing at venues frequented by MSM and TW	Induction	Identified through VBS or referrals from groups that serve PLHIV	Proportion newly tested positive: RR 0.81, 95% CI 0.55−1.18
Njagi M, 2019	Kenya	Quasi‐experimental	497	≥18 years MSM	Recruitment coupons to social and sexual network members	Voluntary counselling and testing at a facility	Induction	MSM enlisted as index recruiters	Proportion newly tested positive: OR 2.18, 95% CI 1.85−2.56
McFall AM, 2018	India	Non‐randomized trial	12,023	≥18 years PWIDs	Recruitment coupons to social network members	HIV testing at a centre for PWID‐focused services	Induction	Two well‐connected PWID selected as seeds	Proportion newly tested positive: IRR 3.98, 95% CI 2.26−7.03
El‐Bassel N, 2022	Kazakhstan	Stepped Wedge CRT	2818	≥18 years PWIDs	Peer‐driven recruitment for HIV testing	HIV testing at the NSPs by walk‐in PWIDs	Induction	Seeds identified by outreach workers	Linkage^b^: IRR 1.14, 95% CI 0.96−1.36
Garofalo R, 2022	Nigeria	Non‐randomized trial	1386	15−24 years young men	Social media for HIV testing and peer navigation	HIV surveillance data at study clinics	Induction	Peer navigators were MSM identified	Proportion newly tested positive: OR 3.11, 95% CI 1.38−7.08
Shahmanesh M, 2021	South Africa	Cluster RCT	2683	18−30 years women	Seeds distributed HIVST packs in their social networks	Clinic referral slips (for HIV testing, prevention and care services) and condoms at a facility	Induction	Initial seeds (18−24 years) approached at random in the community	Linkage^b^: IRR 0.64, 95% CI 0.26−1.62
Chang L, 2015	Uganda	Individual RCT	393	≥18 years, care‐naïve adults with HIV	Peer support on pre‐ART adherence and referral to clinic for care	Clinic‐based pre‐ART care	Alteration	Peers chosen based on their pre‐ART adherence and literacy	Linkage^b^: RR 1.09, 95% CI 1.00−1.19
Young SD, 2015	Peru	Cluster RCT	500	≥18 years MSM	Peer leader engaging with participants via Facebook to encourage HIV testing	HIV testing provided via clinics and joining a Facebook group (no peer leaders)	Individual then segmentation	Well‐respected MSM among the MSM community	Uptake of testing: aOR 2.61, 95% CI 1.55–4.38; adjusted for baseline covariates^b^
Chanda M, 2017	Zambia	Cluster RCT	965	≥18 years FSW	Direct HIVST secondary distribution (direct arm) Coupons distribution for HIVST facility collection (coupon arm)	Standard facility testing	Induction	Peer leaders chosen by research staff	Uptake of testing: a. Control versus direct arm (RR 1.11, 95% CI 0.98−1.21) b. Control versus coupon arm (RR 1.06, 95% CI 0.92−1.22)
Ortblad K, 2017	Uganda	Cluster RCT	960	≥18 years FSW	Direct HIVST secondary distribution (direct arm) Coupons distribution (HIVST at facility‐coupon arm)	Standard facility testing	Induction	Peer leaders chosen by research staff	Uptake of testing a. Control versus direct arm (RR 1.14, 95% CI 1.07−1.22) b. Control versus coupon arm (RR 1.11, 95% CI 1.04−1.19)
**Social network comparator**									
Sha Y, 2022	China	Quasi‐experimental	205	≥18 years MSM	HIV/syphilis test distribution by indexes to network members	Testing card referral by indexes to network members	Induction	Through ads on social media (WeChat & Blued)	Uptake of testing (77.7% vs. 11.5%)
Pettifor, 2020	South Africa	Individual RCT	287	18−26 years females	HCT/HIVST choice arm: choice of either HIVST kits or coupons to distribute	Referral coupons to network members for facility testing	Induction	Chosen by study staff during home visits	Uptake of testing (RR 1.90, 95% CI 1.52−2.48)
Zhou Y, 2022	China	Individual RCT	309	≥18 years MSM	HIV/syphilis self‐testing secondary distribution with incentives (SD‐M arm) SD‐M + online peer referral (SD‐M‐PR arm)	Standard HIV/syphilis self‐testing secondary distribution	Induction	Through ads on social media (WeChat)	Uptake of testing a. Control versus SD‐M‐PR arm (RR 1.11, 95% CI 1.02−1.21) b. Control versus SD‐M arm (RR 1.06, 95% CI 0.96−1.16)

Abbreviations: aOR, adjusted odds ratio; ART, anti‐retroviral therapy; CI, confidence interval; CRT, cluster randomized trial; FSW, female sex worker; HCT, HIV counselling and testing; HIVST, HIV self‐testing; IRR, incidence rate ratio; MSM, men who have sex with men; NSP, needle syringe programme; OR, odds ratio; PLHIV, people living with HIV; PWID, people who inject drugs; RCT, randomized controlled trial; RR, risk ratio; TW, transgender woman; VBS, venue based sampling.

^a^ Linkage: initiating ART for those who tested positive or offer to initiate PrEP for those who tested negative.

^b^ Covariates: age, income, education, ethnic origin, marital status, sexual orientation, computer ownership, time spent daily online and time spent communicating with prospective sexual partners in the past 3 months.

### 3.3 Social network intervention

In the included studies, social networks were either used to accelerate standard testing (at a health facility or prescribed venue by a healthcare worker) or HIVST, and subsequent linkage to care among network members. The commonly employed strategy involved the initial recruitment and training of index participants or seeds who were then tasked with delivering HIV services or invitations to network members (alters). The index participants often shared similar characteristics with the target population (e.g. MSM seeds were used to recruit other MSM). In some cases, the seeds worked with known peers and in other cases with unknown peers, that is no prior existing ties, and they were referred to as “peer leaders” or “peer navigators” or “peer educators” [36, 37, 38, 39]. Various approaches were used to identify the seeds including random selection by the study staff, referrals from organizations working with the target population and adverts on social media (Table 1). The training of the index participants ranged from a brief discussion (about the study, on how to use HIVST kits, on how to recruit network members) to longer training sessions lasting over a day (discussing information about HIV, pre‐ and post‐testing counselling)—Table S2.

The SNIs took on various forms. In some studies, the seeds were delivering invitation coupons to network members to access HIV services at a facility [40, 44, 46, 47], while in others, they were directly delivering the HIVST kits to network members face‐to‐face in the community or via mail [36, 38, 39, 43, 48]. One of the HIVST kit delivery studies used kits that combined testing for HIV and syphilis [48]. Two additional studies used social media outreach: here peer leaders used platforms such as Facebook to disseminate information encouraging network members to take up HIV testing services [37, 45]. The remaining study involved the provision of social support by a peer leader to engage in HIV care [41].

Networks were used to expedite recruitment (either using coupons or direct referrals) or the delivery of a service (distributing HIVST kits or peer support). Education regarding HIV testing and treatment was delivered in combination with coupons or HIVST kits. All studies implicitly used network information to inform the approach used, but only one study explicitly collected network data (number of alters in the seed's personal network) prior to the selection of seeds [46]. However, most studies had a system of linking the unique seeds to their alters, though this information was not commonly used to construct network maps. Two studies that used respondent‐driven sampling (RDS) constructed recruitment chains including the seeds and their alters [36, 46].

While none of the studies explicitly mentioned using Valente's taxonomy, 12 used one network approach: induction (*n =* 11) and alteration (*n =* 1), while the remaining study used a combination of individual and segmentation approaches (Table 1).

### 3.4 Incentives

One study did not mention the use of incentives either for the seeds or the alters [42]. Eight studies provided incentives to the seeds for each alter that was recruited or linked with the study staff by uploading results online (in cases where the delivery of intervention was via mail) [36, 39, 40, 41, 43, 45, 46, 48]. In some cases, both the seeds and the alters received an incentive for fulfilling a set requirement in the study [37, 44, 47], and in some, the alters received reimbursement for their participation only [38].

### 3.5 Theory

Only four studies mentioned theoretical underpinnings for their intervention: the Kimbrough's model [47], social network theory [40], situated information, motivation, and behavioural skills (sIMB) conceptual framework [41] and a blend of components of diffusion of innovations theory and other psychologically driven theories [37]. However, none of the four studies mentioned how the different constructs of the theory informed the intervention development.

### 3.6 Meta‐analyses

#### 3.6.1 Uptake of HIV testing

**Figure 2:**
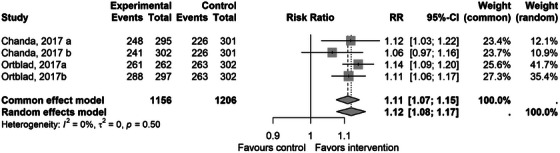
A forest plot showing effect of the social network intervention (experimental) versus non‐network intervention (control) for outcome proportion testing for HIV. CI, confidence interval; RR, risk ratio.

The uptake of HIV testing was reported in five of the included studies [37, 38, 39, 42, 43]. The pooled estimate using eligible studies with a non‐network‐based comparator showed that compared to standard testing, SNIs had a modest effect on the uptake of HIV testing after 4 months (RR 1.12 [95% CI 1.08−1.17, *p* = 0.003]). Each study showed a positive effect, except for one arm in a study by Chanda (Figure 2). These studies also measured testing at 1 month, and the pooled estimate showed no difference between the network intervention and control (RR 1.11 [95% CI 0.89−1.37]) (Figure S1).

A study by Young was excluded from the meta‐analysis due to the use of a distinctly different network approach (social‐media led), which contributed to heterogeneity in the outcomes (Figure S2). This study showed positive significant effects RR 2.60 (95% CI 1.51−4.60) relative to standard testing.

Two of the studies had network‐based comparators; a study by Pettifor and one arm of a study by Zhou showed a positive effect towards the intervention, with RR 2.11 (95% CI 1.47−3.02) and RR 1.11 (95% CI 1.02−1.21), respectively [42, 43]. However, the other arm in Zhou's study (secondary distribution and incentives) showed a small positive effect that was not statistically significant, with RR 1.06 (95% CI 0.96−1.16) [43].

#### 3.6.2 Newly diagnosed cases

**Figure 3:**
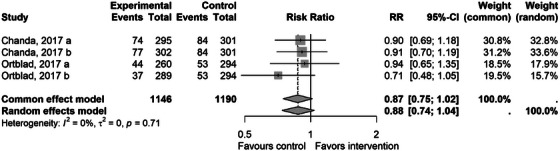
A forest plot showing effect of the social network intervention (experimental) versus non‐network intervention (control) for outcome of the proportion of newly diagnosed cases. CI, confidence interval; RR, risk ratio.

Four studies reported data on HIV testing results [38, 39, 43, 44, 45, 46, 47]. These studies reported newly diagnosed HIV cases following testing. The pooled estimate using eligible studies with a non‐network‐based comparator was RR 0.88 (95% CI 0.74−1.04, *p* = 0.093)—Figure 3. The confidence interval includes 1 and the trend suggests a potential benefit of the control compared to the SNI. Upon reviewing the initial pooled analysis, the study by McFall, 2018 was identified as having a disproportionately large influence on the combined effect size and heterogeneity RR 2.78 (95% CI [2.38, 3.24]) and was, therefore, excluded from the pooled estimate [44, 45, 47].

A three‐arm RCT with a network‐based comparator showed a null effect in both intervention arms (RR 1.16 [95% CI 0.72−1.84] and RR 1.51 [95% CI 0.94−2.43]) [43].

#### 3.6.3 Linkage to HIV care

**Figure 4:**
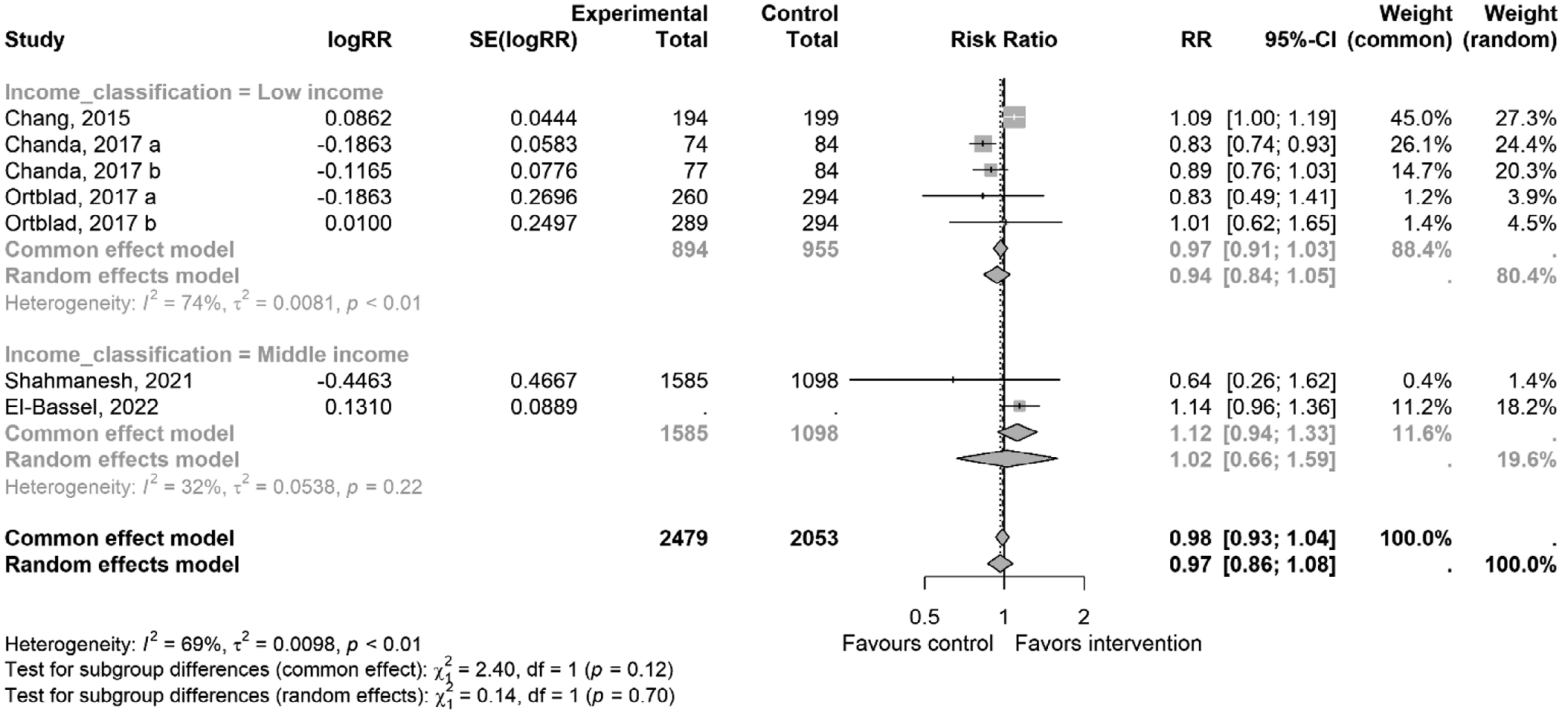
A forest plot showing effect of the social network intervention (experimental) versus non‐network intervention (control) for the outcome of linkage to HIV care grouped by income stratification. CI, confidence interval; RR, risk ratio.

There were five studies that reported data on linkage to HIV care (i.e. initiating ART for those who tested positive and offer to initiate PrEP for those who tested negative) [36, 38, 39, 40, 41]. Only one study reported on PrEP linkage [36]. The pooled estimate using eligible studies with a non‐network‐based comparator was RR 0.97 (95% CI 0.86−1.08, *p* = 0.705)—Figure 4. The directionality of effect was towards the null for most of the studies except for the ones by Chang, 2015 and El‐Bassel, 2022.

### 3.7 Subgroup analyses

Due to the limited number of studies, subgroup analyses were only done for the uptake of testing and linkage to care outcome. A limited number of comparisons were possible (Table 2). The intervention approach refers to the approach that was used to accelerate HIV services in the intervention arm and grouped into two approaches: coupons and direct delivery of HIVST. Direct HIVST showed significant positive effects, while coupons did not show statistically significant effects. Income classification refers to the Gross National Income classification of the country where the study was conducted. The effect on linkage to care did not differ between LMICs (Table 2).

**Table 2:** Subgroup analysis for HIV testing outcome

Subgroup	Number of studies	RR (95% CI)	*Q* value	*p* value for heterogeneity
**Uptake of testing outcome**				
**Intervention approach**			2.33	0.1269
Coupons	2	1.10 (0.86−1.42)		
Direct HIVST	2	1.14 (1.02−1.28)		
**Linkage to care outcome**				
**Income classification**			0.14	0.7048
Low income	5	0.94 (0.84−1.05)		
Middle income	2	1.02 (0.66−1.59)		

### 3.8 Publication bias

The funnel plot (Figure S3) and Egger's test did not provide statistically significant evidence of publication bias (*p* = 0.276).

### 3.9 Quality assessment

Of the eight randomized trials, five had a low risk of bias, one CRT had a high risk of bias, and one CRT and one RCT had some concerns. All the non‐randomized studies were assessed using the ROBINS‐I tool; four of the studies had a serious risk of bias with concerns in the bias for confounding domain. The details of the assessment can be found in Figures S4–S6.

## 4 DISCUSSION

The findings of this systematic review show that SNIs in LMICs predominantly target key populations and exhibit important variability in their design. The meta‐analysis further showed that SNIs had heterogenous effects on the detection of new HIV infection and subsequent linkage to care. However, a modest effect on the uptake of HIV testing was observed which was statistically significant. This, to our knowledge, is the first systematic review and meta‐analysis providing evidence towards the effectiveness of social network HIV testing and linkage interventions in LMICs.

Our results are consistent with other reviews that show that SNIs mainly target at‐risk populations and also effectively increase HIV testing uptake among key populations [49]. However, other reviews demonstrated that SNIs show no positive effects on selected outcomes such as linkage to care [17, 49, 50, 51]. Some individual studies included in this review showed strong positive effects in increasing testing uptake and yield [37, 42, 44, 45]. McFall et al. showed an increase in HIV yield [44]. In this study, PWIDs were recruited using RDS—a targeted approach that probably led to the identification of people who were unreached by conventional methods and a shared high risk of infection. Young et al. targeted MSM through social media outreach and saw a high uptake of HIV testing (RR 2.60 95% CI 1.51−4.50) [37]. The observed positive effects could be because testing becomes more pertinent if one's partner, friend or risk‐sharing friend has tested compared to a random invitation by a healthcare worker to test. Furthermore, with the targeted recruitment that occurs with SNIs, these approaches led to higher identification of undiagnosed cases as individuals tend to cluster together and share similar risky sexual behaviours [44, 45, 52]. Two studies provided weak evidence towards testing uptake [38, 39]. These studies were conducted among FSWs in a high HIV prevalence setting, where information on testing and available testing options were readily accessible. In both studies, peer educators provided support in both the intervention arms and the control and could explain the conservative effect observed. Additionally, FSWs within the same network do not necessarily pose a direct risk of infection to each other, unlike PWIDs who share needles, or MSM who may be sexual partners and thus directly infect one another. Consequently, targeted recruitment could yield higher results among PWIDs or MSM compared to FSWs as observed in the included papers. Additionally, half of the studies that recruited key populations reported positive effects of the intervention on various outcomes [43, 44, 45, 47, 48]. These findings suggest that the approaches employed may have successfully circumvented structural barriers, such as stigma, thereby contributing to the observed effects.

By nature, network interventions target both the index participant and his/her contacts. This is unlike standard trials where the intervention is supposed to benefit the index only. Contamination across arms was highly probable in individual‐level trials or where social media was used, potentially diluting effects. Additionally, where an individual‐level trial design was used, care was supposed to be taken to obtain an unbiased estimate as the independence assumption of observations was generally violated. Cluster RCTs would ideally be the preferred design as they allow for geographical and structural separation of network clusters and should, therefore, minimize contamination.

There were several limitations to this review. First, there was an apparent complexity in clearly reporting what constitutes an SNI. With many variants of SNIs going by different labels and no strict reporting framework, it was challenging to easily identify these interventions. Most papers did not explicitly mention that they were reporting an SNI. In cases where it was implicit, the authors may not have paid attention in accurately describing the social network elements but perhaps focused on the study design elements in the methodology (e.g. reporting elements of an RCT). It took extensive discussions among the review authors or contacting the authors to gain consensus on whether a study truly reported a network intervention. Moreover, this made identifying a consistent and harmonized definition of the treatment across studies included in the meta‐analysis challenging and raises concerns about the comparability of studies within the meta‐analysis. Second, the use of social networks also varied substantially in the different studies. Networks were used to enhance recruitment (either directly or via coupons), in some cases to deliver HIVST (directly, coupons for access at a facility) or to influence network members to access HIV services. This was done either face‐to‐face or through social media. In some studies, financial incentives were given to enhance engagement in the network arms. Consequently, it was challenging to accurately isolate the effect of the networks alone amidst other active components. Additionally, the varied use of networks may have shaped intervention outcomes in distinct ways making direct comparisons difficult. Third, there was a lack of relational data collected in the studies. Despite some studies utilizing RDS as a network sampling approach or for delivery of HIVST, there was a lack of relational data collection and mapping of RDS chains. Relational data is useful in understanding the social network structure, identifying optimal seeds to accelerate the intervention and tailoring interventions to at‐risk clusters [15, 16, 53]. Fourth, some of the included studies referenced theoretical frameworks, but failed to explain how the constructs of these theories were used in developing the interventions. This omission can limit replicability, making it challenging for other researchers to understand the rationale behind the intervention components and to apply these interventions in different contexts. Fifth, few studies met the inclusion criteria constraining our ability to draw robust conclusions. It was not feasible to explore the influence of culture and social differences on the outcomes. Moreover, only limited comparisons were possible for subgroup analysis and some of these had a high risk of bias affecting the validity of our results. As a result, caution is warranted in interpreting the findings, as they may not fully capture the complexity and variability of network interventions across different settings. Lastly, the development‐based boundary was applied in this review to align with our objective. However, we acknowledge that social processes driving the effectiveness of network interventions might transcend these classifications.

This review highlights the need for greater detail in reporting SNIs (harmony in terminology use, use of theories, description of network data). It also provides strong evidence supporting the effectiveness of SNIs in enhancing HIV testing uptake, making them a valuable tool in the global HIV response. Notably, in 2023, for the first time, more new HIV acquisitions were reported outside of SSA [1]. In regions such as eastern Europe, central Asia, Latin America, Middle East and North Africa, where more new acquisitions are being reported, the epidemic is concentrated among key populations [1]. Therefore, targeted interventions, such as those that employ network approaches, are critical to achieving the UNAIDS 95‐95‐95 targets [1].

In settings with widespread testing, SNI designs need to be supported by epidemiological data to help target unreached populations and thus ensure they effectively enhance testing uptake, improve yield and strengthen linkage to care. To maximize their impact, there is a need for policy frameworks that support the integration of SNIs into national HIV strategies. Additionally, incorporating social media into SNI designs can amplify their outcomes as demonstrated in some studies reviewed, though its use can be limited in places where internet access is low. Some studies in this review used social media for advertisement and initial recruitment of participants and had positive effects on various outcomes [37, 43, 48]. However, the residual stigma surrounding an HIV diagnosis and criminalization of key populations in some settings highlights a need to address privacy and confidentiality concerns when designing these interventions.

## 5 CONCLUSIONS

In conclusion, this review summarizes the key design features of network interventions for HIV and their effects on HIV testing and linkage. There is a need for more studies that are designed to capture the complex relational dynamics of SNIs and to generate strong evidence on their isolated effects on some outcomes. Additionally, it is necessary to expand the use of social network approaches to other priority populations as the current focus has been on key populations. These approaches are a vital element of a comprehensive HIV response.

## COMPETING INTERESTS

The authors report no competing interests.

## AUTHORS’ CONTRIBUTIONS

MM, MN, GH, AP, KF and ATC were involved in the conception of the review. MM, HHT, TCM and RS screened and extracted data. MM, HHT, TCM, RS, MN, GH, AP, KF and ATC were involved in planning the analysis. MM and MN performed the analysis. All the authors were involved in interpreting the data. MM wrote the first draft. All the authors read and approved the final version of the manuscript.

## FUNDING

Research reported in this publication was supported by Helse Nord RHF (Grant number: 2019/995) and by the Fogarty International Center of the National Institutes of Health under Award Number D43 TW0010060‐01.

## DISCLAIMER

The content is solely the responsibility of the authors and does not necessarily represent the official views of the National Institutes of Health. The funders had no role in study design, data analysis, decision to publish or preparation of the manuscript.

## Supporting information




**File S1**: Table S1 Detailed search strategy.

**File S2**: Table S2 Characteristics of studies meeting the inclusion criteria.

**File S3**:
**Figure S1**: A forest plot showing effect of the social network intervention (experimental) groups versus non‐network intervention compared with (control) for outcome proportion testing for HIV testing at 1 month.
**Figure S2**: A forest plot showing effect of the social network intervention (experimental) versus non‐network intervention (control) for outcome proportion testing for HIV.
**Figure S3**: A funnel plot on the effect of network interventions on uptake of HIV testing.
**Figure S4**: Traffic plot for cluster randomized trial quality assessments.
**Figure S5**: Traffic plot for individually randomized trial quality assessments.
**Figure S6**: Traffic plot for non‐randomized trial for intervention quality assessments.

## Data Availability

The data supporting the conclusions of this article are available upon request from the corresponding author.
