# Supplementary material for: The key design features and effectiveness of social network interventions for HIV testing and linkage services in low‐ and middle‐income countries: a systematic review and meta‐analysis
Source: J Int AIDS Soc. 2025 Apr 25;28(5):e26458. doi: 10.1002/jia2.26458 (PMC12031894; doi:10.1002/jia2.26458)
Supplement: Supplementary file 2 — File S2: Table S2 Characteristics of studies meeting the inclusion criteria. [file JIA2-28-e26458-s001.docx]

**Supporting File 2**

**Table 2. Characteristics of studies meeting the inclusion criteria**

| **First Author, Year** | **Country** | **Study Design** | **Population** | **Intervention** | **Comparator** | **Network Approach** | **Promotor Selection** | **Promoter Training (if applicable)** | **Use of Incentives** | **Theory** |
| --- | --- | --- | --- | --- | --- | --- | --- | --- | --- | --- |
| **Non-network-based comparator** | | | | | | | | | | |
| Pines H.A, 2021 | Mexico | quasi experimental | ≥18 years old, cisgender male or transgender female, anal sex with a cisgender male or transgender female in the past four months, no previous HIV diagnosis | Recruitment coupons to social networks of MSM and TW by selected seeds to access testing | Targeted recruitment and testing at venues frequented by MSM and TW in Tijuana | Induction | Identified through VBS or referrals from organizations that serve PLWH and selected throughout the study period | Staff helped participants in RDS group to develop peer outreach plans BUT no clear information on training of seed | US$5 per peer referred | Not mentioned |
| Njagi M, 2019 | Kenya | quasi-experimental | ≥18years, self-identified as men, at least 3 Months since last HIV-negative or unknown HIV status; gay, bisexual identity or sex with another male in their lifetime | Social and sexual network index testing (SSNIT)- Recruitment coupons to social & sexual network members | traditional voluntary counselling and testing at a facility | Induction | MSM recently diagnosed with HIV enlisted as index recruiters and HIV positive & HIV-negative enlisted as recipient recruiters | Recruiters (both Index and Alters) were given brief information about the study and coached (through role plays) on how to recruit social network members | USD $3- for study participation & USD $5 for each recipient successfully recruited | Kimbrough’s model |
| McFall A.M, 2018 | India | Non-randomized trial | ≥18years, self-reported injection drug use in the prior 24 months | Recruitment coupons to social network who inject drugs and HIV testing at study site | HIV testing at an Integrated Care Centre (a centre for PWID-focused services-HIV services, Opioid management | Individual then induction | 2 well-connected PWID selected as seeds in each city. Any eligible recipients were also recruited to distribute coupons | Not described | US$3.8- for study participation & US $0.80 for each recipient that enrolled in the study | Not mentioned |
| El-Bassel N, 2022 | Kazakhstan | Stepped Wedge CRT | ≥18 years, People who inject drugs residing in the intervention cities | Peer driven recruitment for HIV testing, HIV counselling and testing, enhanced ART and access | HIV testing at the NSPs by walk-in PWIDs during the periods before the intervention | Induction | Recruiting PWIDs were identified by outreach workers (eligible recipients were also approached to be recruiters) | Outreach workers trained peer recruiters on HIV testing (duration of the training not specified) | $2 for each unique referral | Social Network Theory |
| Garofalo R, 2022 | Nigeria | Non-randomized trial | HIV tests of young men aged 15-24yo, residing in Ibadan city and surrounding areas *study used deidentified data | Social media for HIV testing outreach and peer navigation | HIV surveillance data at study clinics | Induction | Peer navigators were MSM identified | Peer navigators received training on HIV Testing for key pops and how to operationalise the intervention via social media | Peer navigators were incentivised (amount not mentioned) | Not mentioned |
| Shahmanesh M, 2021 | South Africa | CRT | 18-30 years, residing in Africa Health Research Institute (AHRI) demographic surveillance area in uMkhanyakude, KZN | 1. Incentivised peer network (IPN): 18-24yo recruited as seeds to distribute 5 HIVST packs in their social networks  2. Peer navigator direct distribution: HIVST packs direct distribution to participants | Clinic Referral slips (for HIV testing, prevention & care services) & condoms | Induction | Initial seeds (18-24 yo) approached by study staff (peer navigators) at random in the community | A brief demonstration of HIVST kit use | Yes, IPN arm-the original individual (seed) who handed out the coupon received US$1.5 | Not mentioned |
| Chang L, 2015 | Uganda | RCT | ≥18 years, recently diagnosed HIV-infected adults (1-4 weeks), care-naïve | Peer support on Pre-ART adherence, counselling and referral to clinic for care | Clinic-based Pre-ART care | Alteration | Peers chosen based on their Pre-ART adherence and literacy | Peers received a 2-day residential training | $10/Month for peer supporters and $1/participant visited | "situated information, motivation, and behavioral skills  (sIMB) conceptual framework" |
| Young S D, 2015 | Peru | CRT | ≥18years, male, sex with another man in the past 12 months, had a Facebook account or willing to set one up | Peer leader engaging with participants via Facebook to encourage prevention and HIV testing | Enhanced SOC: HIV prevention and testing services provided via local clinics and joining a Facebook group (with no peer leaders) | Individual then Segmentation- Facebook groups | Peer leaders were MSM identified by staff as well respected among the MSM community and interested in educating others about health | Three training sessions of 3 h each were provided for the peer leaders | $14/week for peer leaders and $10 + $14 for participants completing the baseline and endline survey respectively | blended intervention incorporates components of diffusion of innovations theory and other psychologically driven theories |
| Chanda M, 2017 | Zambia | CRT | ≥18 years, FSW, self-reported an HIV-uninfected status | 1. Direct HIVST distribution to network members  2. coupons distribution to network members | Standard facility testing | Induction | Peer leaders chosen by research staff | 2-hour training | $1 per participant | Not mentioned |
| Ortblad K, 2017 | Uganda | CRT | ≥18years FSW | 1. Direct HIVST secondary distribution  2. Coupons distribution (HIVST at facility) | Standard facility testing | Induction | Peer leaders chosen by research staff | peer educators completed a 2-day training | Peer educators received $25/visit- 4 visits | Not mentioned |
| **Network- based comparator** | | | | | | | | | | |
| Sha Y, 2022 | China | quasi-experimental | ≥18 years, male sex assigned at birth and ever had sex with men | HIV/Syphilis test distribution by indexes to network members | Testing Card referral by indexes to network members | Induction | Through ads on social media (WeChat & Blued) | Not described | Yes, $3 for each alter that uploaded results and index | Not mentioned |
| Pettifor, 2020 | South Africa | RCT | 18-26 years females | HCT/HIVST choice arm: choice of either HIVST kits or coupons to distribute | Referral coupons to network members for facility testing | Induction | Chosen by study staff during home visits | Online video on how to self-test | Not mentioned | Not mentioned |
| Zhou Yi, 2022 | China | RCT | ≥18years MSM | 1. HIV/Syphilis Self-testing secondary distribution with incentives (SD-M arm)  2. SD-M + online peer referral (SD-M-PR arm) | Standard HIV/Syphilis Self-testing secondary distribution | Induction | Through ads on social media (WeChat) | Not described | Yes, $3 for each result uploaded (index+ alter). Index received + $3 for each unique alter | Not mentioned |

Abbreviations: CRT: Cluster Randomized Trial; RCT: Randomized Controlled Trial; ART: Anti-retroviral Therapy; VBS: Venue based sampling; FSW: Female Sex Worker; PWID: People who inject drugs; MSM: Men who have sex with Men; TW: Transgender woman; HIVST: HIV self-testing; HCT: HIV Counseling and Testing; NSP: Needle Syringe Program; PLHIV: People living with HIV; RDS: Respondent Driven Sampling ; SOC: Standard of Care; IPN: Incentivized Peer Networks; SD-M: Secondary distribution and monetary incentives; SD-M-PR: Secondary distribution, monetary incentives and peer referral
