# Supplementary material for: The key design features and effectiveness of social network interventions for HIV testing and linkage services in low‐ and middle‐income countries: a systematic review and meta‐analysis
Source: J Int AIDS Soc. 2025 Apr 25;28(5):e26458. doi: 10.1002/jia2.26458 (PMC12031894; doi:10.1002/jia2.26458)
Supplement: Supplementary file 3 — File S3: Figure S1: A forest plot showing effect of the social network intervention (experimental) groups versus non‐network intervention compared with (control) for outcome proportion testing for HIV testing at 1 month. Figure S2: A forest plot showing effect of the social network intervention (experimental) versus non‐network intervention (control) for outcome proportion testing for HIV. Figure S3: A funnel plot on the effect of network interventions on uptake of HIV testing. Figure S4: Traffic plot for cluster randomized trial quality assessments. Figure S5: Traffic plot for individually randomized trial quality assessments. Figure S6: Traffic plot for non‐randomized trial for intervention quality assessments. [file JIA2-28-e26458-s002.docx]

**Supporting File 3**


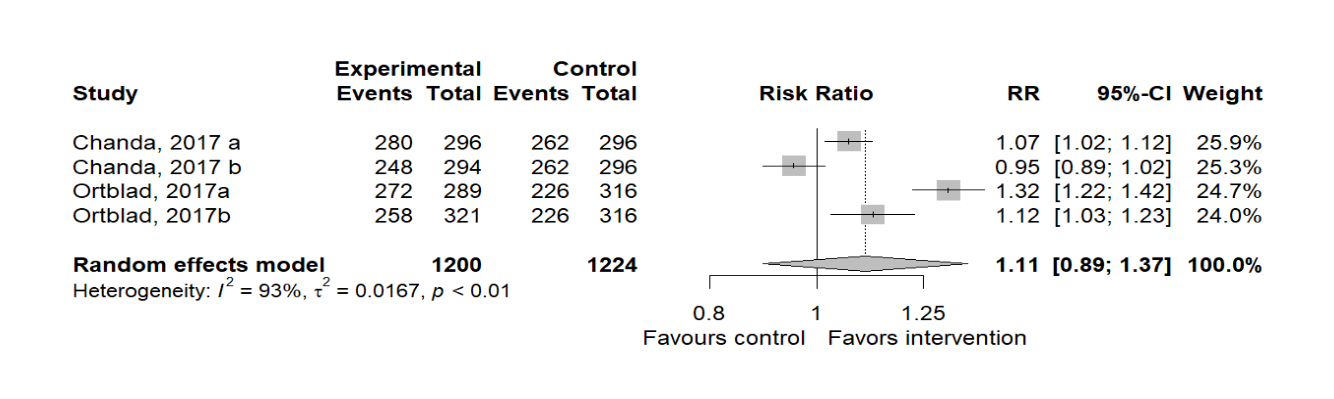


*Figure 1. A forest plot showing effect of the social network intervention (experimental)groups versus non-network intervention compared with (control)s for outcome proportion testing for HIV testing at 1 month*

*Footnote: RR: Risk ratio, CI confidence interval*


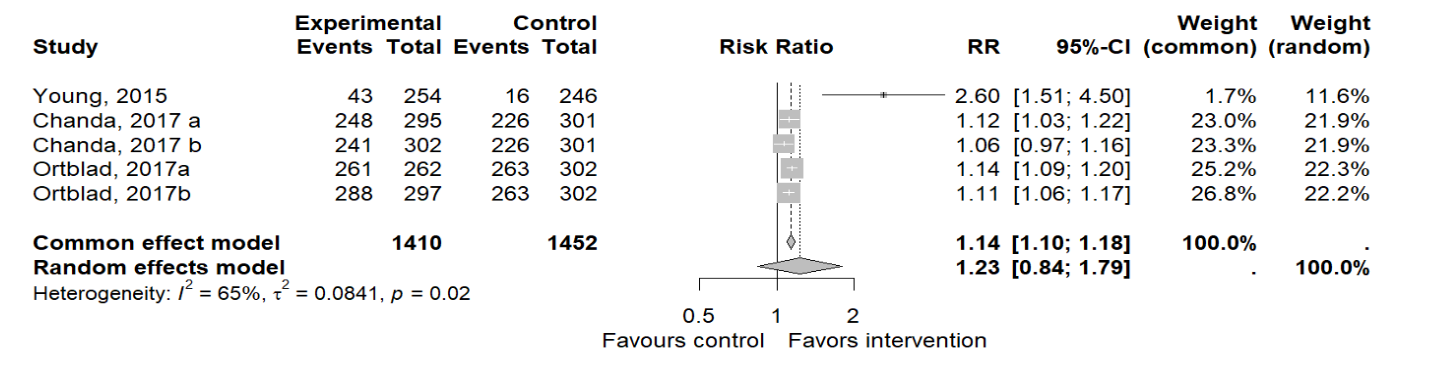


*Figure 2. A forest plot showing effect of the social network intervention (experimental)versus non-network intervention (control) for outcome proportion testing for HIV*

*Footnote: RR: Risk ratio, CI confidence interval*


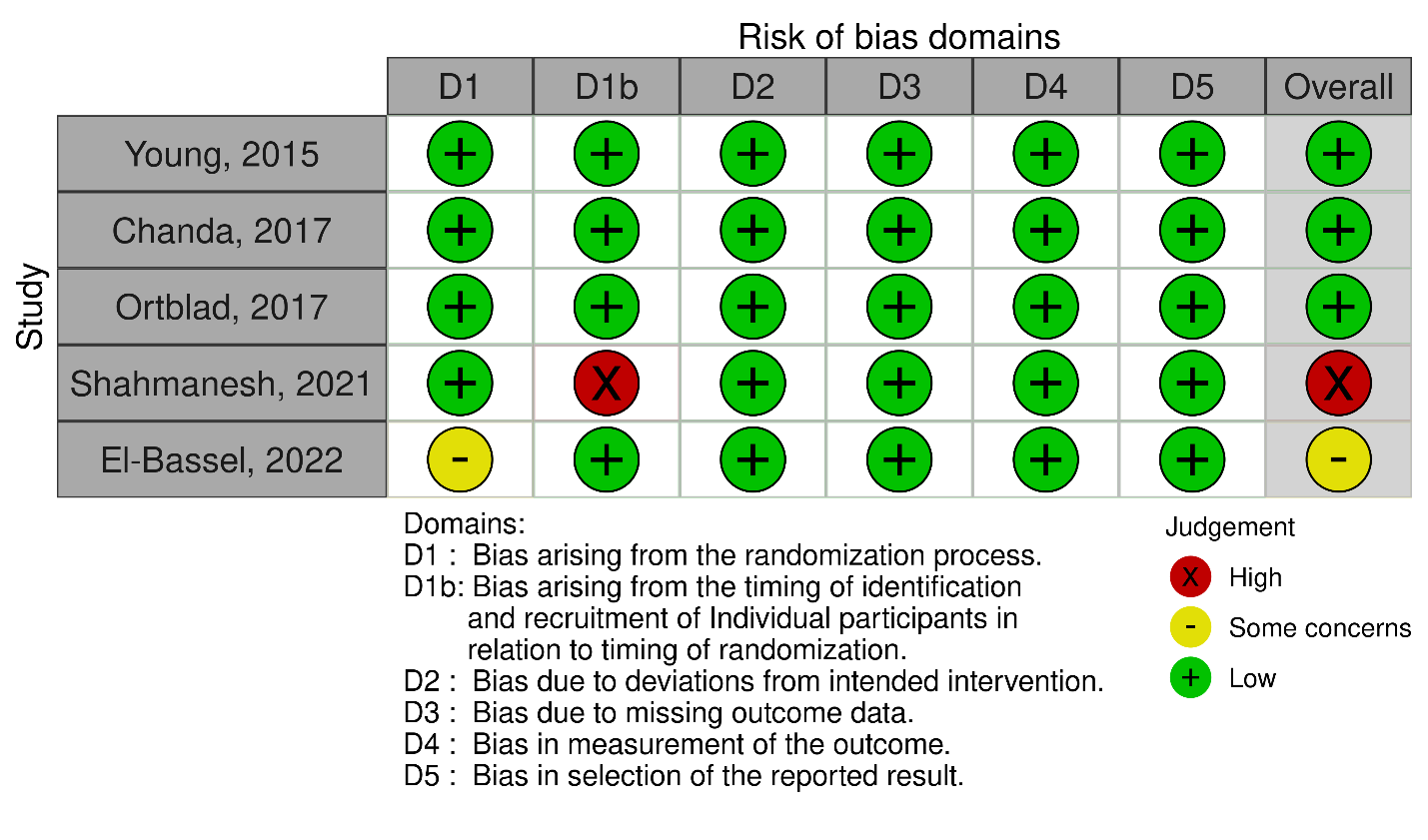


*Figure 3. Traffic plot for Cluster randomized trial quality assessments*


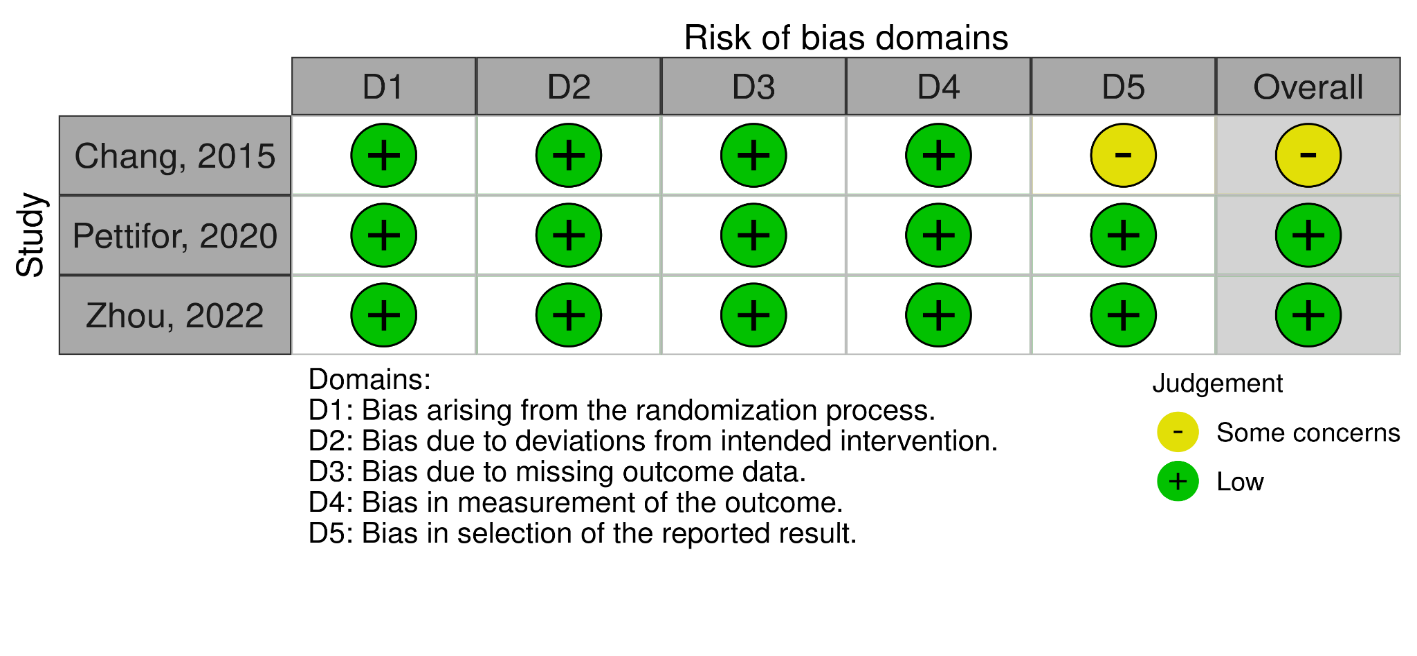


*Figure 4. Traffic plot for individually randomized trial quality assessments*


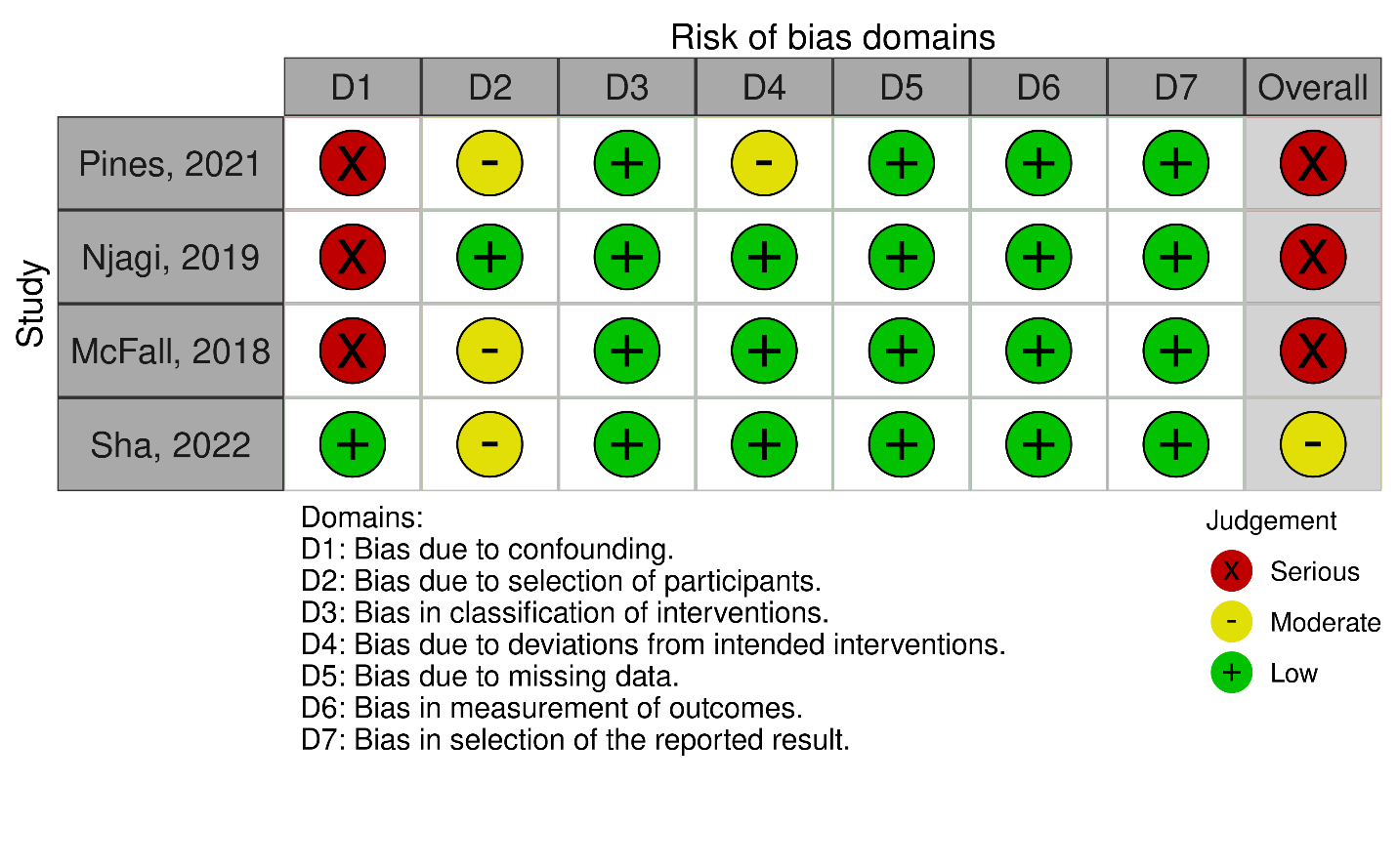


*Figure 5. Traffic plot for Non-randomized trial for interventions quality assessments*


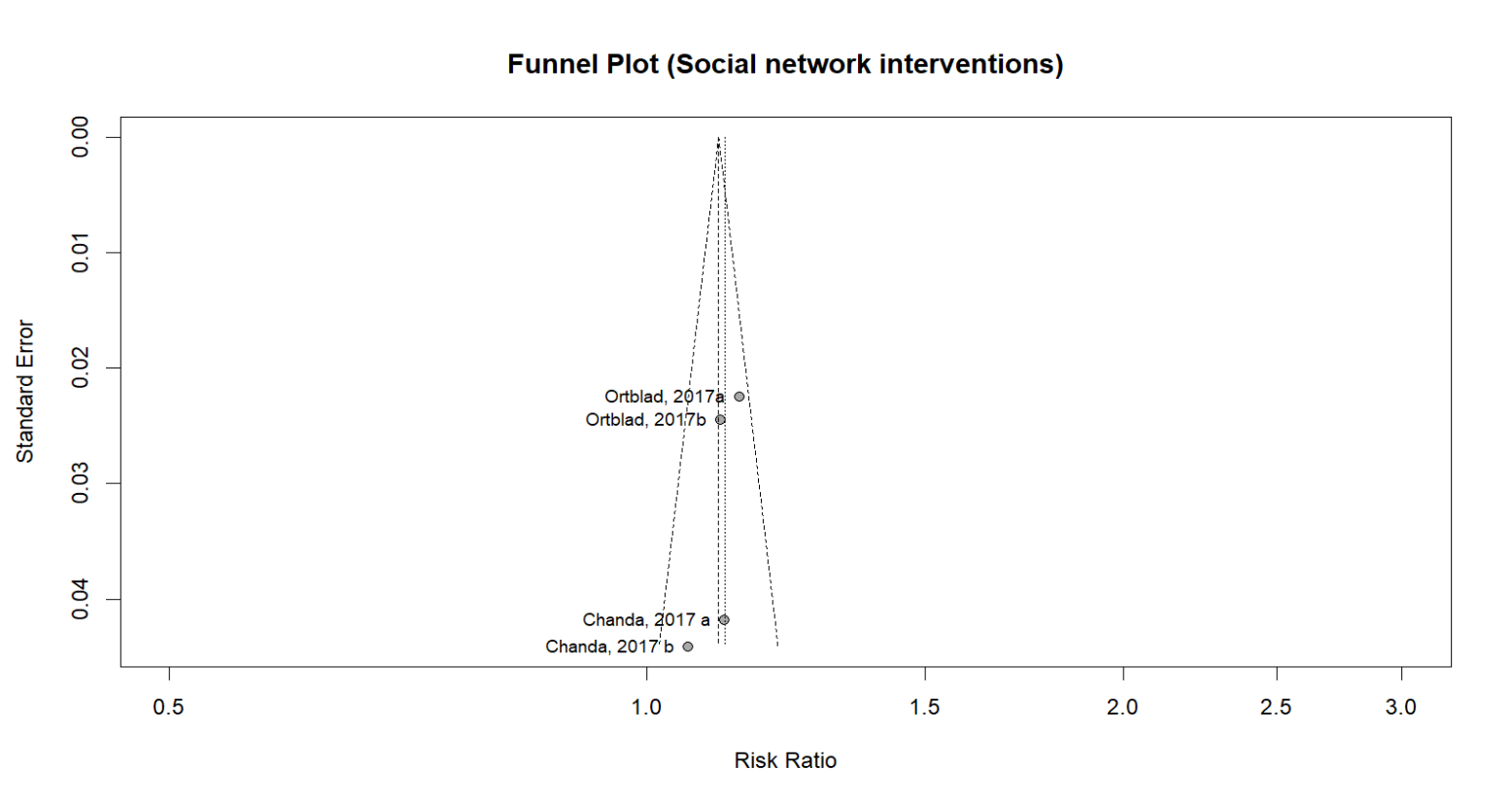


*Figure 6. A funnel plot on the effect of network interventions on uptake of HIV testing*
